# Comprehensive Analysis of the Global Standing of Universities, Research Performance, and the Impact of Scholarly Journals: Pakistan’s Academic and Research Landscape 2026

**DOI:** 10.12669/pjms.42.7.20163

**Published:** 2026-07

**Authors:** Sultan Ayoub Meo, Awaiza Khan, Shaukat Ali Jawaid

**Affiliations:** 1 Sultan Ayoub Meo, MBBS, Ph.D, FRCP Department of Physiology and Clinical Physiology, College of Medicine, King Saud University, Riyadh, Saudi Arabia; 2 Awaiza Khan Department of Biomedical Sciences, Queen Mary University, London, UK; 3 Shaukat Ali Jawaid Chief Editor, Pakistan Journal of Medical Sciences, Karachi, Pakistan

**Keywords:** Pakistan, Universities, Journals, Research, Impact Factor, Citations, Hirsch Index

## Abstract

Academic institutes and innovative research play a pivotal role in knowledge, skills, professionalism, health, economic progress, and the sustainable development of nations. On June 17, 2026, QS World University Rankings released the ranking of global universities. Pakistan has 278 universities, 163 in the public sector and 115 in the private sector. Among these universities, only 02 were ranked in the top 500: Quaid-i-Azam University, Islamabad, ranked 381st, and the National University of Sciences & Technology (NUST), Islamabad, 384th. On the same date, June 17, 2026, Web of Science, Clarivate, released the Journal Citation Report (JCR) of scholarly journals. Worldwide, Clarivate has indexed 22,643 journals from 113 countries. In Pakistan, the Higher Education Commission (HEC) has 723 journals; however, only **34** are indexed in Web of Science: 10 in the Science Citation Index Expanded (SCIE) and 24 in the Emerging Sources Citation Index (ESCI). Pakistan Veterinary Journal achieved an impact factor (IF) of 6.1 and a first-quartile ranking, while Pakistan Journal of Medical Sciences (PJMS) achieved an IF of 1.6 and a second-quartile ranking. In the medical sciences, the highest H-index was achieved by the Pakistan Journal of Medical Sciences (51) and the Journal of the Pakistan Medical Association (44). Pakistan needs to increase educational budget for universities to improve the quality education, research culture and support academic journals in publishing innovative, knowledge-sharing content.

## INTRODUCTION

Academic institutions, universities, higher education, and research are crucial for advancing knowledge, developing skills, and driving human progress. Higher education and research are closely interlinked and play key roles in addressing national and global challenges and in achieving sustainable development for nations. Universities, academic journals, and scientific publications work together to advance science and technology, economic development, and sustainability.^1^-^3^

The impact of universities and education on societal transformation is vital. Universities provide new knowledge and skills to address the challenges of sustainable development in communities by raising public awareness.^4^ In this article, we highlight the global standing of Pakistan’s universities, academic journals, Impact Factor, citations, Hirsch Index (H-Index) and research impact.

### Global standing of Pakistan’s universities:

The university’s global standing is crucial, as it reflects the quality of education, research, and academic reputation. High-ranking universities attract talented students, distinguished faculty, and valuable funding opportunities around the world. Global recognition enhances graduates’ career prospects, increases international partnerships, and promotes innovation. A high global standing helps universities contribute more effectively to society, economic development, and the advancement of knowledge at regional and global scales. University rankings have become a major force in shaping the higher education systems globally.^5^,^6^

On Jun 17, 2026, the World University Rankings, published by the British organization Quacquarelli Symonds (QS),^7^ were released. The QS rankings assess universities based on nine indicators across areas, including academic reputation, research citations, employer reputation, student-to-faculty ratio, proportion of international faculty and students, and international research partnerships.^8^

Pakistan is home to approximately 259.3 million people, the world’s fifth most populous nation.^9^ The country has 278 universities: 163 are in the public sector and 115 are in the private sector. Among these 278 universities, as per the current 2026 QS ranking report,^7^ only 18 universities were ranked: Quaid-i-Azam University, Islamabad, at position 381, and National University of Sciences & Technology (NUST), Islamabad, at position 384 (Table-I). These two universities dropped slightly from their previous year’s positions: Quaid-i-Azam University, Islamabad, from 354 to 381, and National University of Sciences & Technology (NUST), Islamabad, from 371 to 384. (Table-I).

**Table-I:** Global standing of Pakistani Universities and the number of articles published, citations and H-Index.

Name of the University	Rank 2026	Rank 2027	Article published	Times Citations	Highly cited articles	H index
Quaid-i-Azam University, Islamabad	354	381	26995	784937	230	240
National University of Sciences & Technology (NUST) Islamabad	371	384	7202	135270	73	125
Pakistan Institute of Engineering and Applied Sciences, Islamabad	721-730	560	3562	67885	22	95
University of Punjab, Lahore	542	588	30386	622412	281	213
Lahore University of Management Sciences (LUMS)	555	608	3811	75608	32	110
University of Agriculture, Faisalabad	654	629	19454	590459	257	247
COMSATS University Islamabad	664	639	22163	453955	205	180
Government College University, Faisalabad	761-770	691	9210	240778	153	167
University of Engineering & Technology, Lahore	801-850	791-800	1909	35271	10	79
Aga Khan University	1001-1200	951-1000	23693	694205	239	289
University of Peshawar	901-950	951-1000	5572	136392	57	130
The University of Lahore	951-1000	1001-1200	3466	77180	75	109
Bahauddin Zakariya University	1201-1400	1001-1200	11787	279439	137	170
Riphah International University	1201-1400	1201-1400	8106	137434	73	125
University of Karachi	1001-1200	1201-1400	33121	732812	316	261
University of Management and Technology (UMT)	1201-1400	1201-1400	2296	57686	48	101
International Islamic University, Islamabad (IIU)	1201-1400	1400+	4724	122699	62	122
The Islamia University of Bahawalpur, Pakistan	1401+	1401+	12429	250352	148	155

***Note:*** Data was recorded from QS University Ranking 7 and Clarivate, Web of Science.^12^

Pakistan Institute of Engineering and Applied Sciences (PIEAS), Islamabad ranked 560; University of the Punjab, Lahore 588; Lahore University of Management Sciences (LUMS) 608; University of Agriculture, Faisalabad 629; COMSATS University Islamabad 639; Government College University, Faisalabad 691, University of Engineering & Technology (UET) Lahore 791-800; Aga Khan University 951-1000; University of Peshawar 951-1000 (Table-I). The historically renowned and old universities in Pakistan did not secure a position in the top 1000 universities: Bahauddin Zakariya University, Multan, ranked 1001-1200; University of Karachi, ranked 1201-1400; and Islamia University, Bahawalpur, Pakistan, ranked 1400+ (Table-I).

Quaid-i-Azam University, Islamabad, produced 26995 articles with 784937 citations and an H-index of 240. Worldwide, 230 articles were highly cited. Similarly, the National University of Sciences & Technology (NUST), Islamabad, produced 7202 articles and 135270 citations, with an H Index of 125. These two universities ranked among the top 500 universities (Table-I).

The University of Karachi produced 33121articles, received 732812 citations, 316 articles were highly cited, and the H index was 261. Similarly, Aga Khan University, Karachi, produced 23693 articles, received 694402 citations, had 239 highly cited articles, and had an H index of 289. In terms of research productivity, neither the University of Karachi (QS Ranking 1201-1400) nor Aga Khan University (QS Ranking 951-1000) secured the best place in the QS rankings.

Although the University of Karachi and Aga Khan University demonstrate substantial research productivity and visibility, as reflected in their publications, citations, highly cited articles, and H-index, these parameters do not translate into high QS rankings. The QS-Rankings employ a multidimensional methodology that places significant emphasis on academic reputation, employer reputation, faculty-student ratio, internationalization, research impact per faculty member, employment outcomes, and sustainability.^8^ Strong research performance may sometimes be insufficient to achieve a higher QS position when performance on these additional indicators is comparatively weaker. The relatively better QS ranking of Aga Khan University may be attributable to stronger research impact, international collaborations, and institutional reputation despite its lower overall publication volume.

### Functioning of Medical Universities in Pakistan:

It is extremely disappointing that none of Pakistan’s medical universities secured a place among the top 500 universities in the latest international rankings. It is therefore essential to identify the reasons for this poor performance and propose measures to improve.

The functioning of most medical universities, many of which were upgraded from medical colleges, leaves much to be desired. These institutions are not truly autonomous, cannot make independent decisions, and continue to remain under the administrative control of provincial health departments and the federal health ministry. Another issue that frequently comes up for discussion is the loyalty and commitment of both the leadership and the faculty to their respective institutions.

First, the selection of Vice Chancellors should be a merit-based, transparent process and free from any conflict of interest. Second, university leadership and faculty should be able to generate resources by introducing new courses and degree programs, securing research grants, and exploring other funding avenues to strengthen their institutions and avoid remaining dependent on government funding. If suitable candidates are not available within the existing system, there should be no hesitation in thinking outside the box and recruiting talented individuals from outside the current faculty. Such individuals may help improve the image of these universities, establish collaborations with centers of excellence, and build partnerships with leading universities worldwide.

To evaluate the annual achievements of universities, a robust mechanism should be established to assess research funding, grants obtained, publications in impact-factor journals, citations, H-index, and the number of postgraduate students produced across various disciplines.

Pakistani medical universities did not secure positions among the world’s top 500 universities due to limited educational budget, research funding, outdated curricula, limited modern laboratories and facilities, weak industry-academia collaboration, and insufficient collaboration with global universities. Moreover, the bureaucratic hurdles and inconsistent education policies further hinder progress. To improve global rankings, Pakistan should increase investment in higher education, promote research and innovation, strengthen faculty development, update academic programs, encourage international partnerships, improve governance, and enhance industry collaboration. These measures can significantly raise academic quality, research and global competitiveness. A few universities, including Aga Khan University, The University of Lahore, and Riphah International University, are establishing regional and international academic and research collaborations.^10^

### The Landscape of Academic Journals:

Academic journals disseminate knowledge and play a key role in academia, public health, planning, economic progress, and sustainable development. Medical journals are essential pillars of healthcare systems and function as a bridge between scientific research and clinical practice.^11^ The Impact Factor (IF), citations, highly cited articles, and Hirsch Index (H-index) are the most important metrics for evaluating the quality of academic journals. Every year, Clarivate, which operates the Web of Science, releases the Journal Citation Report (JCR).^12^ The JCR provides key information on scholarly journals, including article counts, citation data, quartile rankings (Q1-Q4), impact factors, and H-index. On June 17, 2026, Clarivate announced Journal Citation Report (JCR) 2026, which indexed 22,643 journals from 113 countries across 254 categories. This includes 15,023 science journals, 7,721 social science journals, and 3,460 arts and humanities journals; 6,703 of these are open access, and 521 journals obtain their first IF and secure a place.^12^

### Leading Science Journals:

For the last two decades, the global leading science journals have consistently dominated the academic and scientific landscape across universities and research institutes. The highly worthy journals in the science communities, which achieve high impact factor are the CA-A Cancer Journal for Clinicians, USA with Impact Factor (IF: 685.2), Nature Reviews Molecular Cell Biology, England (IF: 118), The Lancet, England (IF: 109), Nature Reviews Microbiology, England (IF: 104.6), Nature Reviews Drug Discovery, England (IF: 91.2), New England Journal of Medicine, USA (IF: 84.5), JAMA-J American Medical Association (IF: 65.4), Nature, England (IF: 56.1), Nature Medicine, USA (IF: 52.5), Science, USA (IF: 47.3).^12^ These high-impact-factor journals publish peer-reviewed, high-quality research from around the world. They are advanced in scientific fields, attract top researchers worldwide and have a major impact on scientific progress. Their credibility, wide readership, and commitment to excellence make them highly respected publications.

### Academic Journals of Pakistan:

According to the Clarivate Journal Citation Report for academic journals 2026, 34 journals from Pakistan have been indexed in Web of Science.^12^ Among them, 10 are indexed in the Science Citation Index Expanded (SCIE), and 24 in the Emerging Sources Citation Index (ESCI) Table-I. These 24 ESCI journals are still under observation. In the Clarivate Report 2026, five journals achieved an Impact Factor of more than 1. The Pakistan Veterinary Journal, with an IF 6.5 and a quartile ranking (Q1), Pakistan Journal of Medical Sciences IF: 1.6 (Q2), Journal of the College of Physicians and Surgeons Pakistan IF 1.1 (Q3), Pakistan Journal of Agricultural Sciences (IF: 1.3 (Q3), and Pakistan Journal of Botany IF 1.1 (Q3). However, the remaining 05 journals had an IF of less than 1.0 (Table-II).

**Table-II:** Pakistan’s academic journals indexed in Web of Science with Impact Factor and Quartile Ranking.

Name of the Journal	Impact Factor 2024	Quartile Ranking 2024	Impact Factor 2025	Quartile Ranking 2025	Impact Factor 2026	Quartile Ranking 2026
***Journals Indexed in Science Citation Index Expanded (SCIE)***						
Pakistan Veterinary Journal	3.8	Q1	5.4	Q1	6.5	Q1
Pakistan Journal of Medical Sciences	1.2	Q2	1.7	Q2	1.6	Q2
Journal of the Pakistan Medical Association	0.8	Q3	0.8	Q3	0.8	Q3
Journal of the College of Physicians and Surgeons Pakistan	0.7	Q3	0.8	Q3	1.1	Q3
Pakistan journal of agricultural sciences	0.7	Q3	0.6	Q3	1.3	Q3
Pakistan Journal of Botany	0.9	Q4	0.9	Q4	1.1	Q3
Journal of the Chemical Society of Pakistan	0.6	Q4	0.5	Q4	0.6	Q4
Pakistan Journal of Pharmaceutical Sciences	0.7	Q4	0.6	Q4	0.6	Q4
Pakistan Journal of Zoology	0.5	Q4	0.5	Q4	0.5	Q4
Journal of animal and plant Science JAPS	0.6	Q3	0.5	Q4	0.6	Q4
***Journals indexed in Emerging Sources Citation Index (ESCI): Medical Journals***						
Annals of King Edward Medical University Lahore	0.1	Q4	0.1	Q4	0.2	Q4
Pakistan Heart Journal	0.2	Q4	0.2	Q4	0.5	Q4
Rawal Medical Journal	0.4	Q4	0.2	Q4	0.1	Q4
Annals Abbasi Shaheed Hospital Karachi Medical Dental College	0.1	Q4	0.1	Q4	0.1	Q4
Gomal Journal of Medical Sciences	0.5	Q4	0.1	Q4	0.2	Q4
Journal of the Liaquat University of Medical and Health Sciences	0.2	Q4	0.2	Q4	0.2	Q4
Khyber Medical University Journal-KMUJ	0.2	Q4	0.2	Q4	0.3	Q4
Anesthesia Pain & Intensive Care	0.2	Q4	0.3	Q4	0.3	Q4

***Note:*** Data recorded from the Clarivate Report, June 17, 2026.^12^ Total number of SCIE-indexed journals in science and social sciences: 10. Total number of ESCI journals in science and social sciences: 24. This table contains all SCIE 10 journals, and only 08 ESCI-indexed medical journals; the remaining 16 ESCI-indexed science and social journals are not listed.

While analyzing the Emerging Sources Citation Index (ESCI) journals, there are 24 in the sciences and social sciences. Among these 24 journals, 9 are ESCI-indexed medical journals; the remaining 15 are ESCI-indexed science and social sciences journals.^12^ The Annals of King Edward Medical University (AKEMU), Lahore, has an IF of 0.2 and is Q4, and the Khyber Medical University Journal (KMUJ) has an IF of 0.3 and is Q4. These journals are published by the King Edward Medical University, Lahore, and Khyber Medical University, Peshawar, both of which are among the best medical universities in Pakistan. However, AKEMU and KMUJ are still listed in ESCI. Since the last couple of years, AKEMU (IF: 0.1- 0.2) and KMUJ (IF: 0.2-0.3) have been rotating at baseline levels and have not achieved a position in the Science Citation Index Expanded (SCIE). Table-II.

The current Clarivate report demonstrates that among Pakistan’s academic journals, the highest number of articles is published by Pakistan Journal of Botany (72855), Pakistan Journal of Medical Sciences (42924), Journal of the Pakistan Medical Association (35159), and Journal of the College of Physicians and Surgeons Pakistan (26264). Table-III.

**Table-III:** Pakistan’s academic journals with the number of articles, citations, citations per article, number of highly cited articles and H index.

Name of the Journal	First E-JCR Year	Number of articles published	Total citations	Average citations Per item	Highly cited articles	H Index
***Journals Indexed in Science Citation Index Expanded (SCIE)***						
Pakistan Veterinary Journal	2010	2138	18049	8.44	11	42
Pakistan Journal of Medical Sciences	2009	6632	42924	6.47	04	51
Journal of the Pakistan Medical Association	2012	8972	35159	3.92	01	44
Journal of the College of Physicians and Surgeons Pakistan	2009	6521	26264	4.03	00	39
Pakistan journal of agricultural sciences	2012	2066	11080	5.36	01	31
Pakistan Journal of Botany	2007	7941	72855	9.17	03	70
Journal of the Chemical Society of Pakistan	1997	4162	15505	3.73	00	31
Pakistan Journal of Pharmaceutical Sciences	2009	26529	29291	6.26	00	45
Pakistan Journal of Zoology	2007	4349	20036	4.61	00	33
Journal of animal and plant Science JAPS	2020	2027	14345	7.08	00	39
***Medical Journals indexed in Emerging Sources Citation Index (ESCI)***						
Annals of King Edward Medical University Lahore	2020	1635	1743	1.07	00	13
Pakistan Heart Journal	2020	1080	629	0.58	00	7
Rawal Medical Journal	2020	3247	3134	0.97	00	15
Annals Abbasi Shaheed Hospital & Karachi Medical & Dental College	2020	573	299	0.52	00	6
Gomal Journal of Medical Sciences	2020	1062	1755	1.65	00	15
Journal of the Liaquat University of Medical and Health Sciences	2020	1054	1661	1.58	00	11
Khyber Medical University Journal-KMUJ	2020	458	350	0.76	00	6
Anaesthesia Pain & Intensive Care	2020	2032	2252	1.11	00	13

***Note:*** Data recorded from the Clarivate Report, June 17, 2026.^12^

Among Pakistan’s scholarly journals, the highest H-index (a metric that measures the productivity and citations impact) was achieved by Pakistan Journal of Botany (70), Pakistan Journal of Medical Sciences (51), Pakistan Journal of Pharmaceutical Sciences (45), Journal of the Pakistan Medical Association (44), Pakistan Veterinary Journal (42). Table-III

### Appraisal of Impact Factor Rankings Report 2026:

In Pakistan, HEC has 723 journals; however, only 34 are indexed in Web of Science: 10 in SCIE and 24 in ESCI.^12^ Pakistan Journal of Medical Sciences has shown a non-significant decrease of 0.1 in IF from 1.7 in 2025 to 1.6 in 2026. The Journal of the Pakistan Medical Association has maintained an IF of 0.8 in 2025 and 2026. The Journal of the College of Physicians and Surgeons Pakistan have seen an improvement, from 0.7 to 0.8 in 2025 and from 0.8 to 1.1 in 2026 (Table-II).

Academic journals from developing nations often receive lower IF and citation counts due to limited visibility, smaller readership, and research that focuses on local or regional content. Many journals are not indexed in major databases, which further limits the articles’ ability to get citations. Additionally, researchers often prefer to submit high-impact studies to well-established international journals, creating a cycle that favors already prestigious publications. However, lower citation counts do not necessarily indicate lower research quality.^13^,^14^ It is also a fact that, in developing nations, editors work on a part-time honorary basis. There is no adequate infrastructure or support staff. The universities and research institutes must establish a suitable workplace for the journal, and well-trained staff.^15^

The role of regulatory bodies in Pakistan is equally important. The PM&DC and HEC Journals Evaluation and Recognition bodies must foster a culture of scientific publication and improve the standards of academic journals. These regulatory bodies must be on the same page to facilitate the research culture.

Medical universities in Pakistan need to put their house in order. They must be granted genuine independence and autonomy, place greater emphasis on research and publications, attract national and international research grants, and actively pursue collaborations both within the country and abroad. Aga Khan University, the University of Lahore, and Riphah University have achieved success. Improving a university’s global ranking is undoubtedly a challenging task, but it must take the first step in the right direction if they wish to achieve these objectives.

## CONCLUSIONS

In Pakistan, Quaid-i-Azam University, Islamabad, National University of Sciences & Technology, Islamabad, University of the Punjab, Lahore, Aga Khan University, Karachi, University of Agriculture, Faisalabad, University of Karachi, Karachi, University of Peshawar, Peshawar, and COMSATS University, Islamabad contribute a significant volume of research publications and citations. However, only 02 universities, Quaid-i-Azam University, Islamabad, and National University of Sciences & Technology, Islamabad, ranked among the top 500 universities in the global QS ranking. In Pakistan, the Higher Education Commission has 723 journals; however, only 34 are indexed in Web of Science, 10 in the Science Citation Index Expanded, and 24 in the Emerging Sources Citation Index. Pakistan Veterinary Journal and Pakistan Journal of Medical Sciences have earned a worthy place in the global rankings of academic journals. Pakistan must increase the education and research funding for universities and research institutes to foster a culture of quality education, research, and innovation.
